# The Prediction of Stress in Radiation Therapy: Integrating Artificial Intelligence with Biological Signals

**DOI:** 10.3390/cancers16111964

**Published:** 2024-05-22

**Authors:** Sangwoon Jeong, Hongryull Pyo, Won Park, Youngyih Han

**Affiliations:** 1 Department of Health Sciences and Technology, SAIHST, Sungkyunkwan University, Seoul 06355, Republic of Korea; sharkj@skku.edu; 2 Department of Radiation Oncology, Samsung Medical Center, Seoul 06355, Republic of Korea; hr.pyo@samsung.com (H.P.); wonro.park@samsung.com (W.P.); 3 School of Medicine, Sungkyunkwan University, Seoul 06355, Republic of Korea

**Keywords:** radiation oncology, artificial intelligence, biological signals, physiological stress, heart rate variability, machine learning, respiratory irregularity

## Abstract

**Simple Summary:**

Patients undergoing radiation therapy can experience stress because of the fear of treatment. Stress can reduce the accuracy of the patient setup. In this study, we used biological signals to identify the stress response and artificial intelligence to predict this response during radiation therapy. Stress was calculated by analyzing biological signals measured before and during radiation therapy. We used various artificial intelligence models to verify those that were optimized for stress prediction. Our findings indicate that over 90% of patients experience stress during treatment and artificial intelligence can predict this stress with over 80% accuracy. And we validated the impact of stress on respiratory irregularity. This study is pivotal for identifying patients requiring stress reduction before treatment, potentially enhancing the precision of cancer radiation therapy.

**Abstract:**

This study aimed to predict stress in patients using artificial intelligence (AI) from biological signals and verify the effect of stress on respiratory irregularity. We measured 123 cases in 41 patients and calculated stress scores with seven stress-related features derived from heart-rate variability. The distribution and trends of stress scores across the treatment period were analyzed. Before-treatment information was used to predict the stress features during treatment. AI models included both non-pretrained (decision tree, random forest, support vector machine, long short-term memory (LSTM), and transformer) and pretrained (ChatGPT) models. Performance was evaluated using 10-fold cross-validation, exact match ratio, accuracy, recall, precision, and F1 score. Respiratory irregularities were calculated in phase and amplitude and analyzed for correlation with stress score. Over 90% of the patients experienced stress during radiation therapy. LSTM and prompt engineering GPT4.0 had the highest accuracy (feature classification, LSTM: 0.703, GPT4.0: 0.659; stress classification, LSTM: 0.846, GPT4.0: 0.769). A 10% increase in stress score was associated with a 0.286 higher phase irregularity (*p* < 0.025). Our research pioneers the use of AI and biological signals for stress prediction in patients undergoing radiation therapy, potentially identifying those needing psychological support and suggesting methods to improve radiotherapy effectiveness through stress management.

## 1. Introduction

Cancer is a leading cause of death worldwide, and efforts to find successful treatments pose a significant challenge to global health initiatives [1]. Various treatment modalities, such as chemotherapy, immunotherapy, hormonal therapy, surgery, and radiation therapy, are used alone or in combination to treat cancer [2,3]. The role of radiation therapy has been increasing owing to its non-invasive characteristics that are feasible for older patients and the technical advancement of treatment techniques focusing radiation on targeted tumors.

A necessary procedure in radiation therapy involves the creation of an individual treatment plan using simulation computed tomography images to focus high-energy radiation on the tumor while sparing adjacent critical organs. For successful radiation therapy, the accurate positioning of the patient, identical to that in the treatment plan, is essential. If the patient’s position is different from that in the treatment plan, the therapeutic effect is reduced, and damage to normal tissues can occur [4,5,6]. For head and neck cancer, a positioning error of even 3 mm can reduce the dose to the tumor by as much as 10% [7]. Similarly, in cervical cancer, a rotational error of 1° can result in a 2% reduction in the tumor dose and an 11% increase in the dose to adjacent organs at risk [8]. Therefore, accurate beam alignment in accordance with the treatment plan is critical for optimizing therapeutic outcomes.

The accuracy of radiation treatment is influenced by both technological and human factors [9]. Technological uncertainties encompass mechanical issues with radiation therapy equipment, such as imprecision in the leaf position, beam output, and beam profiles, which are typically addressed through regular quality assurance procedures [10,11]. Human factors, such as respiratory and gastrointestinal motion, shrinkage of the targeted tumor volume, and patient stress (anxiety) play significant roles [12,13,14]. The inherent variability in human respiratory and digestive system movements can cause unpredictable displacements of the body and internal organs, which can be mitigated using techniques such as gating, tumor tracking, and image-guided radiation therapy [15]. A treatment plan was modified using adaptive radiotherapy to accommodate the shrinkage of the target volume [16,17]. Although various methods to counteract these factors are used in daily patient treatment and are under development, there is a noticeable scarcity of research addressing the impact of patient stress on radiation therapy.

Psychological stress triggers the sympathetic nervous system, leading to physiological changes such as increased heart rate (HR), blood pressure, breathing rate, and muscle stiffness [18,19,20,21]. Assessment of stress levels in the general population is commonly conducted through surveys [22,23], and this methodology extends to studies examining stress in patients undergoing medical treatment [24]. During the pandemic, the decline in mental health and quality of life of patients with cancer was assessed through a survey [25], and the stress of patients with benign prostatic hyperplasia was confirmed using this same method [26]. He et al. [27] evaluated the influence of anxiety survey responses on treatment setup errors in patients receiving radiation therapy and showed that high anxiety at the initial treatment session tended to result in high setup errors. In other words, stress can impair the accuracy of the radiation therapy setup. Although survey research can efficiently yield data, the reliability of self-reported information is a subject of concern [28]. Consequently, a growing body of research has focused on the measurement of stress through biological signals, which may offer more objective data points than self-reported surveys.

Evaluation of stress through biological signal monitoring is an emerging and pivotal field of medical research. This approach encompasses a variety of metrics, including photoplethysmogram (PPG), electrocardiogram (ECG), body temperature, respiratory patterns, vocal properties, and electroencephalogram (EEG), each offering unique insights into the physiological manifestations of stress [29,30,31]. Under stress, the sympathetic nervous system triggers an increase in body temperature and alters the respiratory dynamics to a faster and shallower pattern. Vocal attributes change noticeably under stress, typically resulting in higher pitch and greater variability. Additionally, EEG recordings reveal an increase in beta-wave activity during stress. Two of the most significant indicators in this field are PPG and ECG, both of which monitor changes in blood flow. Changes in blood flow are instrumental in determining heart rate variability (HRV), a key metric in stress evaluation [32,33]. The reliability and utility of HRV as a stress measure have been substantiated by comparison with traditional stress surveys [34]. Moreover, the integration of HRV analysis into wearable technologies, such as smartwatches, has opened new avenues for real-time, noninvasive stress monitoring [35]. Hence, HRV analysis is a promising alternative to survey-based methods and offers a more objective and continuous assessment of stress levels.

Stress in patients undergoing radiation therapy has been identified in survey studies [36,37]. It was observed that a majority of these patients experience heightened stress levels, particularly in the initial stages of their treatment. This underscores the need for effective stress-management strategies. However, implementing universal stress-reduction measures for all patients can be resource-intensive and requires additional manpower and time. To address this challenge, we leveraged artificial intelligence (AI) techniques in conjunction with biological signal analysis to identify patients who are susceptible to stress during radiation therapy. Our approach involved training machine learning models on HRV data collected both before and during the treatment sessions. This study aimed to use before-treatment HRV data to predict the likelihood of patients experiencing significant stress during therapy sessions. Additionally, we investigated the influence of measured stress on clinical parameters. For that purpose, the correlation of the calculated stress score with irregularities in patients’ respiration was assessed. This prediction enables us to tailor stress management interventions more effectively by focusing on those who need them the most.

## 2. Materials and Methods

### 2.1. Patients

**Figure 1:**
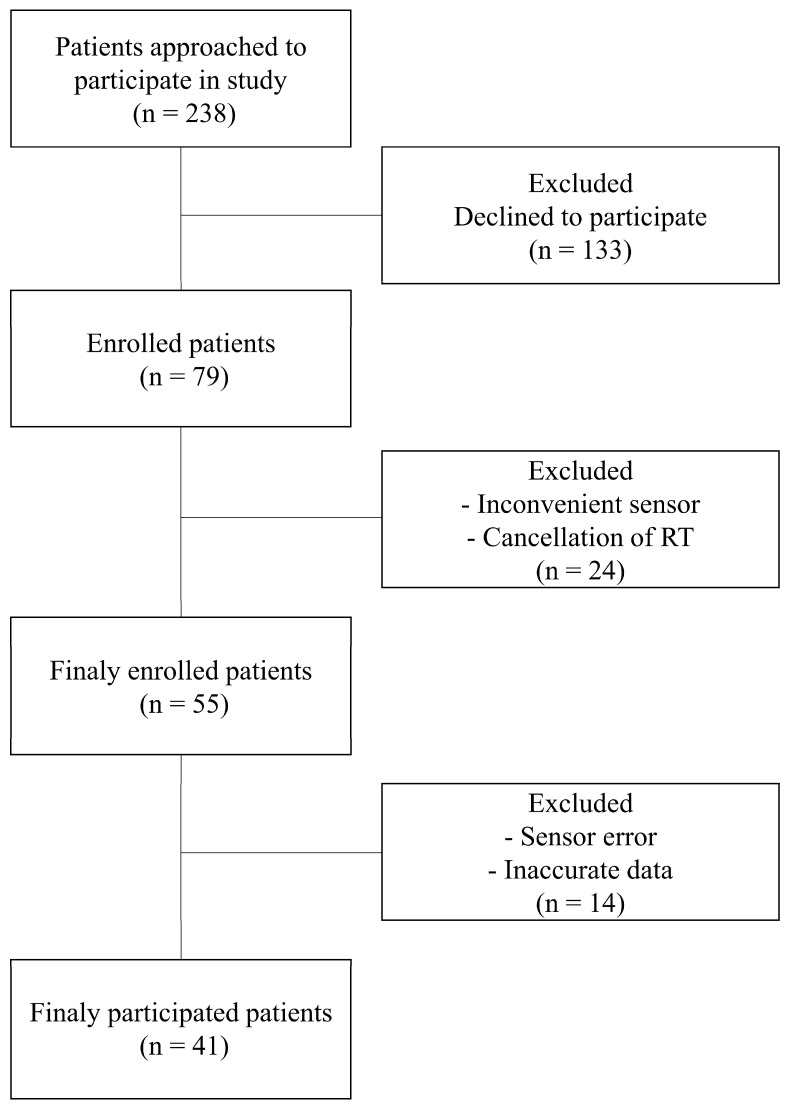
Flowchart of patient enrollment.

The study protocol, including patient recruitment and data collection methods, was approved by the Institutional Review Board of the Samsung Medical Center (IRB number 2020-11-162). Prior to enrollment, written informed consent was obtained from all participants, confirming their voluntary participation and understanding of the study’s aims and processes. Our study prospectively enrolled patients who underwent radiation therapy for lung cancer. The recruitment period spanned from December 2020 to November 2023. The inclusion criteria were carefully defined to ensure a representative and relevant patient cohort. These criteria included (1) adult patients (aged < 80 years) receiving radiation therapy for the first time to capture initial stress responses untainted by previous experiences; (2) patients capable of effective communication, ensuring accurate self-reporting and feedback regarding the study procedures and their well-being; and (3) patients who could comfortably wear the sensor without experiencing discomfort, as any discomfort could confound stress measurements. The patient recruitment process is illustrated in Figure 1. Initially, 238 patients were approached for participation in this study. Of these, 79 consented to participate, reflecting a 33% response rate. During the study, certain patients were excluded due to reasons such as discomfort while wearing the sensor, discontinuation of radiation therapy, or data errors from sensor malfunction. These exclusion criteria helped to maintain the integrity and reliability of the collected data. To ensure the privacy and confidentiality of the participants, all collected data were anonymized. Identifiable information was removed and replaced with unique codes, thereby guaranteeing patient privacy and adhering to ethical data-handling practices.

### 2.2. Data Acquisition and Processing

Data collection commenced with patients wearing a biological sensor (Laxtha, Ubpulse 360, Daejeon, Republic of Korea) upon arrival in the waiting room prior to receiving radiation therapy. The sensor was positioned on the finger to ensure no interference during the treatment procedure. After a 10 min acclimatization period, the patients were escorted to the treatment room where they continued to wear the sensor throughout their radiation treatment session. The radiation treatment time ranged from 16 to 42 min with an average of 27 min. Upon completion of the treatment, the sensor was returned, and the collected PPG data were securely transferred to a dedicated computer system for analysis. Signals arising from patient movements and those resulting from sensor errors were carefully removed to ensure data integrity. To analyze stress changes during treatment, a minimum of 1 day and a maximum of 5 days of data were extracted for each patient. Subsequently, the PPG data were segmented into two distinct phases for analysis: the before-treatment phase, captured while the patient was in the waiting room, and the during-treatment phase, recorded when the patient was lying on the treatment couch. To account for potential HR elevations due to movement, we isolated 5 min of data following a 2 min stabilization period in both the before- and during-treatment phases. From these phases, the HRV was computed by analyzing the intervals between successive PPG peaks. Preprocessing of the PPG data and subsequent HRV analyses were conducted using MATLAB R2020b (MATLAB, MathWorks, Natick, MA, USA) to ensure a standardized and reproducible methodology.

### 2.3. Stress Features

Identification and accurate quantification of stress features are important for the assessment of stress levels using HRV analysis. In this study, we operationalized stress using a set of physiological markers derived from PPG signals. The second derivative of the PPG signal was used to pinpoint the heartbeat peaks, and HRV was calculated by measuring the intervals between these peaks. The selection of HRV-related stress features was based on a comprehensive literature review, identifying seven features consistently associated with physiological stress responses [38,39,40,41,42,43,44,45,46,47,48]. These features were HR, standard deviation of normal-to-normal (NN) intervals (SDNN), square root of the mean sum of squares of successive NN interval differences (RMSSD), percentage of successive NN intervals differing by more than 50 ms (pNN50), power of high-frequency range (HF), ratio of low-frequency range/high-frequency range (LF/HF), and total power of frequency range (TP). Under stable conditions, stress was typically indicated by increased HR and LF/HF, whereas SDNN, RMSSD, pNN50, HF, and TP decreased (Table 1). We employed these stress features to calculate the stress score (range: 0−100%) by observing changes before and during treatment.

### 2.4. Stress Prediction

**Figure 2:**
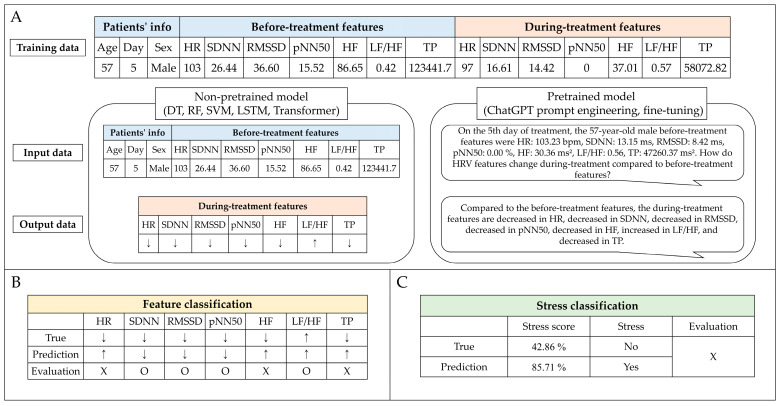
Stress prediction workflow using artificial intelligence. (**A**) The training process of the non-pretrained and pretrained models. (**B**) Feature classification evaluation process. (**C**) The stress classification evaluation process based on 50% of the stress score calculated using the results of feature classification. ↑: increase; ↓: decrease; O: done; Χ: not done; HR: heart rate; SDNN: standard deviation of normal-to-normal (NN) intervals; RMSSD: square root of the mean sum of squares of successive NN interval differences; pNN50: percentage of successive NN intervals differing by more than 50 ms; HF: power of high-frequency range; LF/HF: ratio of low-frequency range/high-frequency range; TP: total power of frequency range.

Predicting patient stress in the waiting room before treatment is crucial to enhance the accuracy of preparing patients for radiation treatment. This enables the early implementation of measures to reduce stress, potentially improving treatment efficacy. Non-pretrained and pretrained models were used for stress prediction. The non-pretrained model categories included decision tree (DT) [49], random forest (RF) [50], support vector machines (SVM) [51], long short-term memory (LSTM) [52], and transformer [53]. The pretrained models used were OpenAI’s ChatGPT, which is based on a large language model (LLM) and enables prompt engineering and fine-tuning. Prompt engineering involves the strategic design of input prompts to elicit the desired responses from an LLM [54], whereas fine-tuning refers to the process of adjusting an LLM’s parameters on a specific dataset to improve its performance for particular tasks [55]. The non-pretrained models were assessed using 10-fold cross-validation to evaluate their ability to handle eight different input datasets (Type 1, only before-treatment features; Type 2, before-treatment features with age; Type 3, before-treatment features with sex; Type 4, before-treatment features with day; Type 5, before-treatment features with age and sex; Type 6, before-treatment features with age and day; Type 7, before-treatment features with sex and day; and Type 8, before-treatment features with age, sex, and day). These datasets included treatment day, age, sex, and seven stress features identified before treatment. The model outputs were designed to classify the predicted changes in stress features during treatment (Figure 2). Subsequently, the top three input datasets from the performance of the non-pretrained models were selected for further analysis with the pretrained models. The pretrained model was evaluated against a representative one-fold out of a 10-fold cross-validation of the non-pretrained model. Therefore, the pretrained and non-pretrained models compared the results of the one-fold dataset. The pretrained models performed prompt engineering in GPT-3.5 and GPT-4.0 and fine-tuning in GPT-3.5-turbo-1106.

### 2.5. Evaluation

A comprehensive evaluation of our predictive models involved several statistical and machine learning metrics to assess the stress score distribution and its variation throughout the treatment course. We analyzed the aggregated stress score changes and classified them by sex to observe potential differences in stress patterns between male and female patients over a period of up to four days. The non-parametric Wilcoxon signed-rank test was employed for paired comparisons, whereas the Friedman test was used to analyze changes across multiple-day trends. The Mann–Whitney U test was used to compare stress scores between males and females.

To assess the predicted stress features during treatment, we adopted two analytical approaches: feature classification (multi-label) and stress classification (binary). Feature classification utilized the raw output from our models to evaluate prediction accuracy across multiple labels. The key metrics included the exact match ratio (EMR) and standard classification metrics such as accuracy, recall, precision, and F1 score, providing a holistic view of the models’ performance. The feature classification result was calculated as a stress score, but the stress classification uses the categories of “yes (>50%)” or “no (<50%)” based on the criterion of a stress score of 50%. The effectiveness of the stress classification was quantified using accuracy, recall, precision, and F1 score.

To investigate whether stress measured through biological signals has an impact on clinical parameters, we assessed respiratory irregularities in patients during treatment. We defined respiratory irregularity using the mean of the standard deviations (*STD*) of the peaks and valleys of breathing signals [56].

(1) $$Respiratory\;irregularity\;=\;\frac{STD(peaks)+STD(valleys)}{2},$$

Irregularity Equation (1) was utilized to calculate irregularities in both amplitude and phase. Amplitude irregularity was quantified by its height, while phase irregularity was assessed through period measurements. Our analysis investigated the correlation between respiratory irregularity and predefined stress categories (“yes” or “no”), as well as the increase in stress score. We employed generalized estimating equations to accommodate the correlated structure of the repeated measures data [57].

The non-pretrained models were developed using Python (version 3.7.16) with traditional machine learning algorithms, such as DT, RF, and SVM, implemented via the Scikit-learn library (version 1.3.2). Deep learning algorithms, such as LSTM and transformer, were operationalized using Pytorch (version 1.7.1), and all computations were performed on an NVIDIA GeForce 2080Ti GPU. The Scikit-learn library was utilized to compute various performance metrics to ensure consistency and reliability in our evaluation methodology.

## 3. Results

### 3.1. Patient Characteristics

Our study enrolled 41 patients, comprising 27 males (65.85%) and 14 females (34.15%) with a mean age of 67.15 years (interquartile range: 47.0–80.0 years). Table 2 presents a detailed summary of the characteristics of the enrolled patients, including sex distributions and age ranges. Regarding stress analysis, we observed 123 cases of stress measurements over the course of the study. Of these, 12 were identified without stress indicators, representing a stress-free state. The most frequently observed stress score was 85.71% (*n* = 26). Stress was recorded for up to 14 days. However, from the fifth day onward, the number of stress cases recorded each day was less than 10.

### 3.2. Stress Score Changes as Treatment Progresses

**Figure 3:**
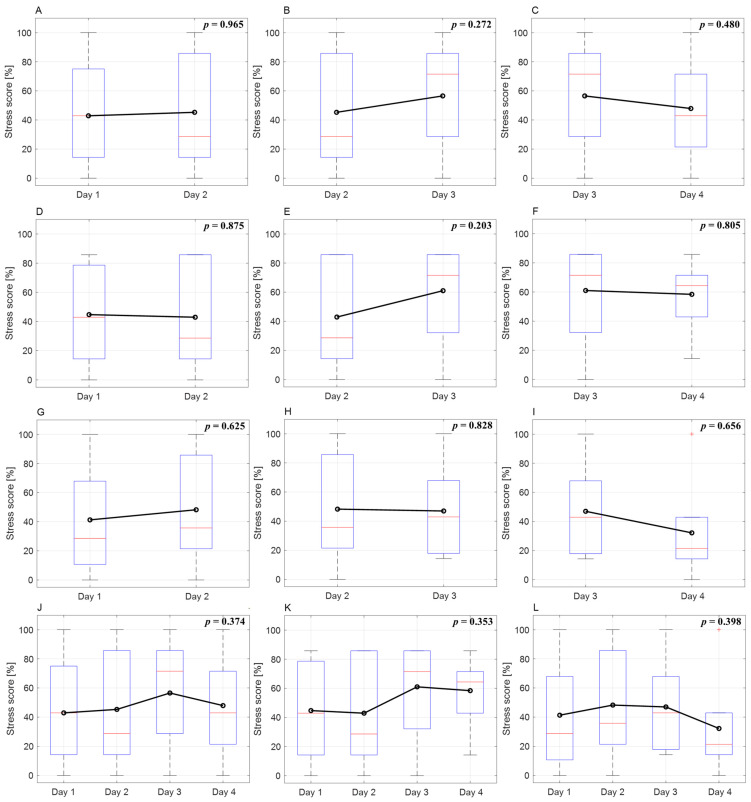
Comparison of stress score changes by date and overall trends. (**A**–**C**) Comparison of changes in the stress score of all patients for each day. (**D**–**F**) Comparison of changes in the stress score of male patients for each day. (**G**–**I**) Comparison of changes in the stress score of female patients for each day. (**J**) Analysis of trends in stress score change for all patients and all dates. (**K**) Analysis of trends in stress score change across all days for male patients. (**L**) Analysis of trends in stress score change across all days for female patients.

Using data from days one to four, when more than 15 stress cases were obtained, we confirmed the change in stress score over time (Figure 3). There were no differences by date or overall trends in the all, male, and female data. Although the trend throughout treatment was not significant, stress scores increased in males and decreased in females. The difference in stress scores between males and females on day four was significant (*p* = 0.0384) (Table 3).

### 3.3. Non-Pretrained Model Features Classification

In our analysis, we utilized stress case data to classify the treatment features using non-pretrained models across eight different input datasets. The average results of the 10-fold cross-validation are summarized in Table 4. In terms of the EMR, the LSTM model using the Type 8 dataset performed the best, achieving an EMR of 0.172. However, the RF model exhibited superior performance across all other datasets. Regarding accuracy, the LSTM model with the Type 7 dataset had the highest accuracy of 0.699. Moreover, the LSTM model had the highest metrics in recall with the Type 6 dataset, reaching a peak value of 0.793. Regarding the precision and F1 scores, the DT model using the Type 1 dataset demonstrated the strongest results.

### 3.4. Pretrained Model Features Classification

Datasets 6, 7, and 8 exhibited the highest scores for recall, accuracy, and EMR, respectively, in the non-pretrained model and were selected for pretrained model evaluation (Table 5). The pretrained model was evaluated only one-fold, and for an intuitive comparison, the non-pretrained model performance shown in Table 5 was a one-fold result selected from the 10-fold data. Across the three datasets, GPT3.5 with prompt engineering did not achieve an accuracy exceeding 0.5. In contrast, GPT4.0 outperformed GPT3.5 in all evaluation metrics. In the Type 8 dataset, the fine-tuned GPT3.5-turbo-1160 model exhibited the most impressive results, whereas its performance in the Type 7 dataset was comparatively lower. When focusing on the Type 7 dataset, the LSTM model achieved the highest accuracy and recall. However, for the Type 8 dataset, GPT4.0 with prompt engineering emerged as a superior model in terms of EMR, precision, and F1 score.

### 3.5. Stress Classification

Stress classification was binary (Figure 2C). The feature classification result is calculated as a stress score, but the stress classification uses the categories of “yes (>50%)” or “no (<50%)” based on the criterion of a stress score of 50%. Therefore, these results differ from those of the feature classification. As shown in Table 6, among the non-pretrained models, the LSTM using the Type 7 dataset achieved the highest accuracy (0.846) in stress classification. For the pretrained models in the Type 8 dataset, GPT4.0 with prompt engineering demonstrated the highest accuracy at 0.769, and GPT3.5 with prompt engineering had the highest recall at 0.800 but the lowest accuracy at 0.385. The RF and SVM models exhibited equivalent performances across the three types of datasets. The DT and transformer models were the most effective for the Type 8 dataset.

### 3.6. Respiratory Irregularity

**Figure 4:**
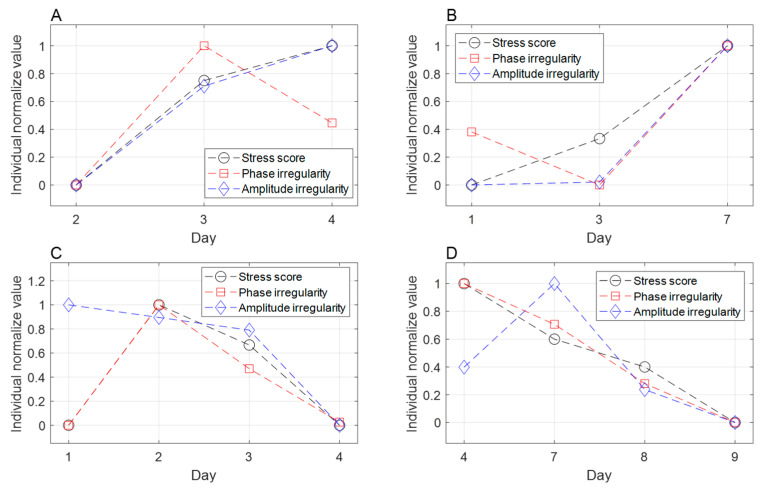
Trends in respiratory irregularities and stress scores over the course of treatment. The stress scores are normalized to the maximum score for each patient. (**A**,**B**): changes in stress scores are similar to amplitude irregularity. (**C**,**D**): changes in stress scores are similar to phase irregularity.

Among the patients who participated in the study, 77 cases of respiratory irregularity were calculated from 27 patients (18 males and 9 females, average age 67.91) whose respiratory signals were monitored using AN RPM respiratory gating system (Varian Medical System, Palo Alto, CA, USA) during radiation therapy. The average phase irregularity was 10.060 with a minimum of 3.354 and a maximum of 29.651. The average amplitude irregularity was 0.143 with a minimum of 0.086 and a maximum of 0.370. As shown in Table 7, a 10% increment in stress and a high stress score (>50%) were both significantly correlated with phase irregularity, while the correlation with amplitude irregularity did not reach statistical significance. Figure 4 visualizes the similarity between the breathing irregularity increments and the stress scores.

## 4. Discussion

Stress in patients undergoing radiation therapy can lead to muscle stiffness, which can affect the accuracy of treatment setup and potentially cause accidents due to movement or falls. Although posttreatment surveys have validated stress in patients undergoing radiation therapy, in-room stress during treatment remains unmeasured. Our study utilized biological signals and found that 90% of patients experienced stress during treatment. Our research enables the identification of cancer patients undergoing radiation therapy who require interventions to reduce stress before treatment. By recognizing and mitigating stress in advance, the accuracy of radiation therapy can be enhanced, ultimately improving treatment outcomes.

Table 2 presents the distribution of the during-treatment stress scores measured using biological signals from 41 patients. Of the 123 stress cases, 12 (9.76%) showed no stress, while 111 (90.24%) indicated stress. The highest stress score distribution (85.71%) was observed in 26 patients (21.14%). The evaluation of the presence of stress based on a 50% stress score threshold was 47.15%. Stiegelis et al. [58] found that 21−54% of patients undergoing radiation therapy experienced stress. This range is reflective of our findings; that is, using a 50% stress score as a threshold, we observed that 47.15% of cases experienced stress.

Figure 3 shows the variation in patients’ stress scores over different days. For males, the stress scores on days 1 and 2 were similar, exceeding 50% on day 3 and remaining similar on day 4. In females, there was a slight increase on day 2, a decrease on day 3, and a significant decrease on day 4. Overall, except for day 2, males exhibited higher stress scores than females on all dates. Furthermore, males exhibited an increasing trend in stress as treatment progressed, whereas females showed a decreasing trend. However, trends in stress score changes were not statistically significant. Irwin et al. [59] indicated that female stress decreased over the course of treatment, whereas male stress did not change significantly. Although not statistically significant, our study’s stress score trends showed tendencies similar to those of other research findings.

Implementing pre-treatment measures to reduce stress is challenging for all patients. When calculating stress using a threshold of a 50% stress score, 47.15% of the cases exhibited a stress response. Antoni et al. [36] and Irwin et al. [59] found that factors such as age, occupation, marital status, and sex differences do not significantly affect stress. While our study found higher initial stress in females, the overall stress scores were higher in males. Considering the referenced studies and our research, it may be inaccurate to select specific patient groups for before-treatment stress-reduction measures. Therefore, it is necessary to predict stress in all patients prior to treatment.

Our study utilized five non-pretrained models and eight dataset types to classify changes in the features during treatment (Table 4). The RF model exhibited the best overall EMR across the datasets, and the LSTM model had the highest EMR of 0.172 for the Type 8 dataset. The LSTM performed best in terms of accuracy across all datasets, particularly in the Type 7 dataset, with an accuracy of 0.699. Similarly, LSTM had the highest recall across all datasets. The DT model had the highest precision and F1 scores of 0.683 and 0.639, respectively. In predictive modeling, accurately identifying actual stress states is crucial, rather than mislabeling non-stressed individuals as stressed. Hence, the Types 6, 7, and 8 datasets exhibited the highest recall, accuracy, and EMR, respectively, and were selected to evaluate the pretrained model using one-fold data.

In the analysis presented in Table 5, for the Types 6 and 7 datasets, the LSTM model continued to outperform the others in terms of EMR, accuracy, and recall, which is consistent with the findings shown in Table 4. However, in the Type 8 dataset, both GPT4.0 and LSTM demonstrated superior performance in EMR, achieving a score of 0.231. While LSTM led to accuracy and recall, GPT4.0 excelled in precision and F1 score. The GPT3.5 model displayed the lowest performance across all indicators in these datasets, with GPT3.5-turbo-1160 achieving an accuracy of 0.615 for the Type 8 dataset.

Considering all models, including non-pretrained and pretrained models, the LSTM model demonstrated robust performance across all evaluation indices and datasets, making it the most suitable for feature classification during treatment. In scenarios where implementing a machine learning model is challenging, the pretrained GPT4.0 model, particularly with the Type 8 dataset, emerged as the most appropriate choice.

Stress classification uses the categories of “yes (>50%)” or “no (<50%)” based on the criterion of a stress score threshold of 50% (Table 6). In stress classification, the LSTM of the Type 7 dataset classified stress effectively with an accuracy of 0.846. The RF and SVM models exhibited a stability of 0.769 accuracy across all datasets. For the pretrained model, GPT4.0 showed an accuracy of 0.769 in the Type 8 dataset that included all data, but in the Type 7 dataset, all pretrained models failed to exceed the accuracy of 0.5. As with feature classification, LSTM was the best among all models for stress classification, with GPT4.0 being superior for the Type 8 dataset. The GPT4.0 model is suited for predictions using diverse patient information, whereas LSTM is recommended because of its stability in scenarios with limited information.

Datasets 6, 7, and 8, which are used for comparison in Table 5 and Table 6, contain the treatment days. The treatment day is important information for stress prediction. The performance of the non-pretrained model was similar across the three dataset types. However, the pretrained model’s performance was the best in the Type 8 dataset, which included age, sex, and treatment day, and the worst in the Type 7 dataset, in which age was omitted. Stress prediction using a pretrained model may be better when using all available patient information.

Our study is a pioneer in the use of before-treatment information to predict during-treatment stress, in contrast to most studies that have focused on current stress. Gazi et al. [60] predicted stress in surveys using biological signals such as respiration, ECG, and electrodermal activity and showed an accuracy of 86%, and Vulpe-Grigorași et al. [61] used ECG and neural networks to predict survey stress with 85% accuracy. A few studies have predicted future stress levels. Clark et al. [62] used the driver’s breathing, ECG, and galvanic skin response signals in real time to predict stress after 1 min with 94% accuracy. Taylor et al. [63] used signals such as the participant’s 24 h physiology, weather, number of calls, and location to predict the next day’s mood with an accuracy of 82.2%. Although a direct comparison with these studies is difficult, in our study, the LSTM using the Type 7 dataset showed an accuracy of 84.6%. The accuracy of our research in predicting future information and HRV information obtained through limited PPG was sufficiently high, and we believe that the addition of learning datasets and patient biological signals will result in even higher accuracy.

To investigate the potential impact of stress on radiation therapy, we analyzed stress influences on respiration irregularity during treatment delivery (Table 7). Phase irregularity exhibited a significant increase in the stressed group compared to the non-stressed group, with an estimated mean difference of 2.191 (*p* < 0.017). A 10% rise in stress score was correlated with a 0.286 increase in phase irregularity (*p* < 0.025). Although stress also showed a tendency to elevate amplitude irregularity, the difference did not reach statistical significance. Phase-based gated radiotherapy relies on the consistency of the respiratory cycle, and irregular respiratory can compromise treatment accuracy and prolong delivery time [64]. Our findings demonstrate a significant association between stress and respiratory irregularity, suggesting that stress management could enhance treatment accuracy and precision for radiation therapy patients undergoing gated delivery.

This study had certain limitations. This study focused on patients with lung cancer who underwent their first radiation therapy session. The use of finger-worn sensors did not affect therapy for patients with lung cancer. However, the limited number of methods for measuring biological signals and the narrow patient population resulted in restricted participant diversity and a lack of standardization in stress assessment methods. Expanding the research to include various cancer patients using sensor technologies that do not interfere with treatment could enhance the accuracy of stress prediction and enable more precise evaluations. Although AI-based stress prediction using biological signals has demonstrated over 80% accuracy, the impact of the measured stress score on the actual radiation therapy remains insufficiently validated. Although a correlation between elevated stress and respiratory irregularity has been established, further research is required to analyze the correlations between stress indicators and variables related to treatment accuracy, such as setup error, and setup times. Nevertheless, the correlation between stress and respiratory irregularity suggests that stress may influence radiation therapy outcomes. Assigning weights to features with a high correlation could lead to more accurate stress assessments. Future research will aim to select appropriate sensor technologies and involve diverse cancer patient groups.

The effect of stress on radiation therapy is difficult to quantify and is not clearly understood. To the best of our knowledge, this is the first study to develop a tool to quantify stress in patients undergoing radiation therapy. We demonstrated the validity of the developed method by showing a significant correlation between the stress score and respiratory irregularity in patients. Respiratory irregularity is one of the parameters that exhibits the impact of stress on radiation therapy accuracy.

## 5. Conclusions

This study successfully predicted the stress scores of patients undergoing radiation therapy and demonstrated a correlation between stress and respiratory irregularity. This tool has the potential to improve the accuracy of radiation therapy through the psychological care of patients with high stress. This study will pave the way for understanding patient stress during radiation therapy and exploring its impact on various aspects, thereby providing better care for the success of cancer treatment with radiation beams.

## Figures and Tables

**Table 1:** Summary of stress-related features based on heart rate variability.

Features	Unit	Description	Stressful
HR	bpm	Average number of heart beats per minute	Increase
SDNN	ms	Standard deviation of normal-to-normal (NN) intervals	Decrease
RMSSD	ms	Square root of the mean sum of squares of successive NN interval differences	Decrease
pNN50	%	Percentage of successive NN intervals differing by more than 50 ms	Decrease
HF	ms^2^	Power of the high-frequency range (0.15–0.4 Hz)	Decrease
LF/HF	ms^2^	Ratio of the low-frequency range/high-frequency range	Increase
TP	ms^2^	Total power of the frequency range (0.004–0.4 Hz)	Decrease

**Table 2:** Characteristics of enrolled patients.

Characteristic	N	(%)
All patients	41	(100)
Sex		
Male	27	(65.85)
Female	14	(34.15)
Age		
All	Mean, 67.15	(Range, 47–80)
Male	Mean, 66.56	(Range, 47–80)
Female	Mean, 68.29	(Range, 57–80)
Stress case		
All	123	(100)
Male	81	(65.85)
Female	42	(35.15)
Stress score		
0%	12	(9.76)
14.29%	18	(14.63)
28.57%	18	(14.63)
42.86%	17	(13.82)
57.14%	6	(4.88)
71.43%	17	(13.82)
85.71%	26	(21.14)
100%	9	(7.32)
Stress case day		
1	17	(13.82)
2	18	(14.63)
3	22	(17.89)
4	20	(16.26)
5–14	46	(37.40)

**Table 3:** Stress scores of males and females on each day.

	Day 1	Day 2	Day 3	Day 4
Male	44.69 ± 33.70%	42.90 ± 35.67%	61.01 ± 29.83%	58.39 ± 22.37%
Female	41.31 ± 39.45%	48.26 ± 37.40%	46.99 ± 31.67%	32.17 ± 31.28%
*p*-value	0.8707	0.6691	0.2978	0.0384

**Table 4:** Non-pretrained model features classification.

Dataset	Model	EMR	Accuracy	Recall	Precision	F1 Score
Type 1	DT	0.147	0.638	0.638	**0.683**	**0.639**
	RF	0.163	0.646	0.654	0.645	0.625
	SVM	0.108	0.599	0.593	0.671	0.606
	LSTM	0.115	0.669	0.665	0.487	0.539
	Transformer	0.138	0.628	0.528	0.390	0.412
Type 2	DT	0.123	0.637	0.643	0.669	0.632
	RF	0.165	0.645	0.673	0.616	0.615
	SVM	0.074	0.580	0.577	0.677	0.599
	LSTM	0.156	0.679	0.764	0.537	0.598
	Transformer	0.113	0.631	0.571	0.347	0.394
Type 3	DT	0.148	0.618	0.609	0.677	0.620
	RF	0.165	0.645	0.656	0.612	0.616
	SVM	0.106	0.571	0.559	0.649	0.576
	LSTM	0.156	0.689	0.700	0.501	0.567
	Transformer	0.131	0.617	0.456	0.287	0.323
Type 4	DT	0.115	0.611	0.617	0.665	0.615
	RF	0.164	0.656	0.673	0.640	0.635
	SVM	0.083	0.573	0.575	0.624	0.572
	LSTM	0.132	0.662	0.719	0.499	0.565
	Transformer	0.122	0.624	0.491	0.323	0.362
Type 5	DT	0.106	0.644	0.643	0.663	0.632
	RF	0.147	0.653	0.670	0.641	0.632
	SVM	0.074	0.557	0.542	0.631	0.560
	LSTM	0.124	0.678	0.698	0.496	0.559
	Transformer	0.130	0.610	0.461	0.373	0.394
Type 6	DT	0.107	0.620	0.611	0.679	0.621
	RF	0.147	0.649	0.673	0.604	0.612
	SVM	0.091	0.566	0.566	0.644	0.573
	LSTM	0.115	0.689	**0.793**	0.517	0.604
	Transformer	0.114	0.609	0.486	0.319	0.361
Type 7	DT	0.140	0.615	0.614	0.661	0.614
	RF	0.164	0.651	0.675	0.640	0.631
	SVM	0.090	0.573	0.574	0.645	0.578
	LSTM	0.139	**0.699**	0.776	0.549	0.615
	Transformer	0.131	0.611	0.370	0.348	0.336
Type 8	DT	0.100	0.621	0.612	0.662	0.614
	RF	0.163	0.641	0.656	0.612	0.609
	SVM	0.082	0.554	0.546	0.627	0.558
	LSTM	**0.172**	0.680	0.708	0.487	0.551
	Transformer	0.073	0.611	0.404	0.355	0.344

Underlined values are the best for each dataset, and underlined and bold values are the best overall. Type 1: only before-treatment features; Type 2: before-treatment features with age; Type 3: before-treatment features with sex; Type 4: before-treatment features with day; Type 5: before-treatment features with age and sex; Type 6: before-treatment features with age and day; Type 7: before-treatment features with sex and day; Type 8: before-treatment features with age, sex, and day; EMR: exact match ratio; DT, decision tree; RF, random forest; SVM: support vector machine; LSTM: long short-term memory.

**Table 5:** Non-pretrained and pretrained model features classification.

Dataset	Model	EMR	Accuracy	Recall	Precision	F1 Score
Type 6	DT	0.000	0.637	0.727	0.635	0.672
	RF	0.077	0.637	0.777	0.611	0.669
	SVM	0.077	0.670	0.799	0.646	0.701
	LSTM	0.154	0.681	0.895	0.552	0.655
	Transformer	0.077	0.505	0.552	0.332	0.360
	GPT3.5 (P)	0.077	0.440	0.610	0.330	0.397
	GPT4.0 (P)	0.000	0.615	0.746	0.665	0.674
	GPT3.5-turbo-1160 (F)	0.154	0.527	0.726	0.412	0.503
Type 7	DT	0.077	0.582	0.688	0.593	0.632
	RF	0.154	0.626	0.768	0.585	0.649
	SVM	0.154	0.637	0.783	0.581	0.646
	LSTM	0.154	**0.703**	**0.907**	0.587	0.707
	Transformer	0.000	0.560	0.453	0.563	0.492
	GPT3.5 (P)	0.077	0.396	0.538	0.358	0.416
	GPT4.0 (P)	0.077	0.560	0.667	0.629	0.638
	GPT3.5-turbo-1160 (F)	0.000	0.484	0.593	0.512	0.538
Type 8	DT	0.000	0.637	0.727	0.635	0.672
	RF	0.154	0.626	0.768	0.585	0.649
	SVM	0.077	0.648	0.780	0.599	0.660
	LSTM	**0.231**	0.692	0.781	0.635	0.685
	Transformer	0.000	0.560	0.375	0.571	0.451
	GPT3.5 (P)	0.077	0.407	0.369	0.278	0.246
	GPT4.0 (P)	**0.231**	0.659	0.742	**0.731**	**0.723**
	GPT3.5-turbo-1160 (F)	0.077	0.615	0.714	0.613	0.646

Underlined values are the best for each dataset, and underlined and bold values are the best overall. Type 6: before-treatment features with age and day; Type 7: before-treatment features with sex and day; Type 8: before-treatment features with age, sex, and day; EMR: exact match ratio; DT, decision tree; RF, random forest; SVM: support vector machine; LSTM: long short-term memory; (P): prompt engineering; (F): fine-tuning.

**Table 6:** Non-pretrained and pretrained model stress classification.

Dataset	Model	Accuracy	Recall	Precision	F1 Score
Type 6	DT	0.615	0.375	0.429	0.400
	RF	0.769	0.400	0.571	0.471
	SVM	0.769	0.400	0.571	0.471
	LSTM	0.692	0.444	0.500	0.471
	Transformer	0.538	0.571	0.400	0.471
	GPT3.5 (P)	0.385	0.600	0.300	0.400
	GPT4.0 (P)	0.615	0.500	0.444	0.471
	GPT3.5-turbo-1160 (F)	0.615	0.375	0.429	0.400
Type 7	DT	0.615	0.250	0.400	0.308
	RF	0.769	0.400	0.571	0.471
	SVM	0.769	0.400	0.571	0.471
	LSTM	**0.846**	0.364	**0.667**	0.471
	Transformer	0.538	0.571	0.400	0.471
	GPT3.5 (P)	0.462	0.500	0.333	0.400
	GPT4.0 (P)	0.385	0.200	0.167	0.182
	GPT3.5-turbo-1160 (F)	0.462	0.167	0.200	0.182
Type 8	DT	0.692	0.333	0.500	0.400
	RF	0.769	0.400	0.571	0.471
	SVM	0.769	0.400	0.571	0.471
	LSTM	0.769	0.400	0.571	0.471
	Transformer	0.692	0.000	0.000	0.000
	GPT3.5 (P)	0.385	**0.800**	0.333	0.471
	GPT4.0 (P)	0.769	0.300	0.600	0.400
	GPT3.5-turbo-1160 (F)	0.615	0.250	0.400	0.308

Underlined values are the best for each dataset, and underlined and bold values are the best overall. Type 6: before-treatment features with age and day; Type 7: before-treatment features with sex and day; Type 8: before-treatment features with age, sex, and day; EMR: exact match ratio; DT, decision tree; RF, random forest; SVM: support vector machine; LSTM: long short-term memory; (P): prompt engineering; (F): fine-tuning.

**Table 7:** Analysis of the influence between stress and respiratory irregularity.

Irregularity	Stress	Beta	95% LCL	95% UCL	*p*-Value
Phase	Stress score 10%	0.286	0.036	0.536	0.0247
	Stress “yes” or “no”	2.191	0.399	3.982	0.0166
Amplitude	Stress score 10%	0.003	−0.001	0.007	0.1111
	Stress “yes” or “no”	0.018	−0.006	0.042	0.1323

Stress score 10%: a 10% rise in stress score. Stress “yes” or “no” refers to categories of “yes (>50%)” or “no (<50%)” determined by a 50% threshold in stress score for classification. Beta: the increase in respiratory irregularity associated with a 10% increase in the stress score or the differentiation between “yes” or “no” stress categories. LCL: lower confidence limit. UCL: upper confidence limit.

## Data Availability

The datasets used in this study are available from the corresponding author upon reasonable request.
